# Enlarged Perivascular Spaces in Infancy and Autism Diagnosis, Cerebrospinal Fluid Volume, and Later Sleep Problems

**DOI:** 10.1001/jamanetworkopen.2023.48341

**Published:** 2023-12-19

**Authors:** Dea Garic, Robert C. McKinstry, Joshua Rutsohn, Rebecca Slomowitz, Jason Wolff, Leigh C. MacIntyre, Leigh Anne H. Weisenfeld, Sun Hyung Kim, Juhi Pandey, Tanya St. John, Annette M. Estes, Robert T. Schultz, Heather C. Hazlett, Stephen R. Dager, Kelly N. Botteron, Martin Styner, Joseph Piven, Mark D. Shen

**Affiliations:** 1 Carolina Institute for Developmental Disabilities, University of North Carolina at Chapel Hill School of Medicine, Chapel Hill; 2 Department of Psychiatry, University of North Carolina at Chapel Hill School of Medicine, Chapel Hill; 3 Mallinckrodt Institute of Radiology, Washington University School of Medicine, St Louis, Missouri; 4 Department of Biostatistics, Gillings School of Global Public Health, University of North Carolina at Chapel Hill, Chapel Hill; 5 Department of Psychology, University of Denver, Denver, Colorado; 6 Department of Educational Psychology, University of Minnesota Twin Cities College of Education and Human Development, Minneapolis; 7 McGill Centre for Integrative Neuroscience, Montreal Neurological Institute-Hospital, McGill University, Montreal, Canada; 8 Center for Autism Research, Children’s Hospital of Philadelphia, University of Pennsylvania Perelman School of Medicine, Philadelphia; 9 Department of Speech and Hearing Science, University of Washington, Seattle; 10 University of Washington Autism Center, University of Washington, Seattle; 11 Department of Radiology, University of Washington Medical Center, Seattle; 12 Department of Psychiatry, Washington University School of Medicine in St Louis, St Louis, Missouri

## Abstract

**Importance:**

Perivascular spaces (PVS) and cerebrospinal fluid (CSF) are essential components of the glymphatic system, regulating brain homeostasis and clearing neural waste throughout the lifespan. Enlarged PVS have been implicated in neurological disorders and sleep problems in adults, and excessive CSF volume has been reported in infants who develop autism. Enlarged PVS have not been sufficiently studied longitudinally in infancy or in relation to autism outcomes or CSF volume.

**Objective:**

To examine whether enlarged PVS are more prevalent in infants who develop autism compared with controls and whether they are associated with trajectories of extra-axial CSF volume (EA-CSF) and sleep problems in later childhood.

**Design, Setting, and Participants:**

This prospective, longitudinal cohort study used data from the Infant Brain Imaging Study. Magnetic resonance images were acquired at ages 6, 12, and 24 months (2007-2017), with sleep questionnaires performed between ages 7 and 12 years (starting in 2018). Data were collected at 4 sites in North Carolina, Missouri, Pennsylvania, and Washington. Data were analyzed from March 2021 through August 2022.

**Exposure:**

PVS (ie, fluid-filled channels that surround blood vessels in the brain) that are enlarged (ie, visible on magnetic resonance imaging).

**Main Outcomes and Measures:**

Outcomes of interest were enlarged PVS and EA-CSF volume from 6 to 24 months, autism diagnosis at 24 months, sleep problems between ages 7 and 12 years.

**Results:**

A total of 311 infants (197 [63.3%] male) were included: 47 infants at high familial likelihood for autism (ie, having an older sibling with autism) who were diagnosed with autism at age 24 months, 180 high likelihood infants not diagnosed with autism, and 84 low likelihood control infants not diagnosed with autism. Sleep measures at school-age were available for 109 participants. Of infants who developed autism, 21 (44.7%) had enlarged PVS at 24 months compared with 48 infants (26.7%) in the high likelihood but no autism diagnosis group (*P* = .02) and 22 infants in the control group (26.2%) (*P* = .03). Across all groups, enlarged PVS at 24 months was associated with greater EA-CSF volume from ages 6 to 24 months (β = 4.64; 95% CI, 0.58-8.72; *P* = .002) and more frequent night wakings at school-age (*F* = 7.76; η^2^ = 0.08; *P* = .006).

**Conclusions and Relevance:**

These findings suggest that enlarged PVS emerged between ages 12 and 24 months in infants who developed autism. These results add to a growing body of evidence that, along with excessive CSF volume and sleep dysfunction, the glymphatic system could be dysregulated in infants who develop autism.

## Key Points

**Question:**

Are enlarged perivascular spaces (PVS) in infancy associated with autism diagnosis, cerebrospinal fluid (CSF) volume, and later sleep problems?

**Findings:**

In this cohort study of 311 infants at high or low familial likelihood of autism, infants aged 24 months with high familiar risk and a diagnosis of autism had significantly higher prevalence rates of enlarged PVS than infants with no autism with or without familial risk. Enlarged PVS were also associated with increased CSF volume and later sleep problems.

**Meaning:**

These findings suggest that enlarged PVS could be an early indication of glymphatic dysfunction and that aberrant CSF dynamics may play a role in the development of autism.

## Teaser

This cohort study examines the prevalence of enlarged perivascular spaces in infants who develop autism compared with controls and trajectories of extra-axial cerebrospinal fluid volume and sleep problems in later childhood.

## Introduction

Understanding of the importance of cerebrospinal fluid (CSF) dynamics is rapidly evolving, with emerging evidence indicating that movement of CSF is critical for maintaining brain health. CSF circulates through brain tissue via a recently discovered network of perivascular spaces (PVS), now known as the glymphatic system,^1^ delivering growth factors and removing neuroinflammatory solutes to maintain neural homeostasis.^2,3,4,5,6,7^ PVS (also known as Virchow-Robin spaces) are pial-lined channels that surround small blood vessels in the brain and facilitate the exchange of CSF and interstitial fluid.^8^ PVS play a crucial role in neural waste clearance, with dysfunction associated with certain neurological disorders,^8^ cognitive decline, and neurodegeneration in aging populations.^9,10,11,12,13,14^

During infancy, the brain and CSF system undergo rapid growth and may be particularly vulnerable to impaired CSF dynamics. However, there is a paucity of research examining PVS in children, particularly those with neurodevelopmental disorders. The limited data available suggest that children with enlarged PVS are nearly 5 times more likely to have developmental delay and 12 times more likely to have psychiatric problems.^15^ Studies^16,17^ have suggested that enlarged PVS are more prevalent in children with autism, but these studies are limited by small sample sizes, wide age ranges, and evaluating children after the age of autism diagnosis, leaving open the questions of whether enlarged PVS are evident prior to an autism diagnosis and the potential association of enlarged PVS with the emergence of symptoms.

While PVS have yet to be evaluated in infancy, there is consistent evidence of CSF anomalies in the early development of autism. In 3 previous independent samples, Shen et al^18,19,20^ reported excessive volume of CSF in the subarachnoid space, or extra-axial CSF (EA-CSF), in infants and toddlers as young as age 6 months who were later diagnosed with autism. Accumulation of EA-CSF volume is hypothesized to arise from impaired CSF circulation and to be linked to enlarged PVS, but the association between EA-CSF volume and enlarged PVS has never been investigated in children, to our knowledge. Assessment of PVS could potentially be more scalable to clinical practice, given that PVS are routinely evaluated by radiologists, while EA-CSF requires image processing and segmentation. Sleep problems are common in autism,^21,22,23,24,25^ and sleep helps regulate CSF dynamics and function of the glymphatic system.^26,27,28,29,30,31^ An association between sleep problems and enlarged PVS has been observed in several studies of aging adults,^26,27,28^ but it remains unclear whether enlarged PVS could be developmentally consequential if they are present in early life.

Following on these points, the aims of this study were to (1) determine whether infants who develop autism had higher rates of enlarged PVS than controls, (2) evaluate whether enlarged PVS are associated with EA-CSF volume, and (3) to examine whether enlarged PVS in infancy are associated with later sleep problems at school-age (ie, ages 7-12 years). To address these questions, we analyzed an existing data set of longitudinal magnetic resonance imaging (MRI) acquired from 311 infants and children aged 6 to 24 months^32,33^ who were at higher likelihood of autism diagnosis (HL; ie, with an older sibling diagnosed with autism) or lower likelihood of autism (LL; ie, without an older sibling diagnosed with autism). A subset of participants returned at school-age and were assessed for sleep problems by questionnaire. We hypothesized that (1) infants with HL who were later diagnosed with autism (HL-positive) would have higher rates of enlarged PVS compared with infants with HL who did not develop autism (HL-negative) and infants with LL who did not develop autism (LL-negative), (2) presence of enlarged PVS would be associated with greater EA-CSF volume, and (3) enlarged PVS during in infancy would be associated with sleep problems at school-age.

## Methods

For this cohort study, all study procedures were approved by each site’s institutional review board, and written informed consent was obtained from each participant’s parent. This study is reported following the Strengthening the Reporting of Observational Studies in Epidemiology (STROBE) reporting guideline.

### Participants

This study was conducted as part of the Infant Brain Imaging Study (IBIS), an ongoing multisite longitudinal study that collects behavioral and neuroimaging data on infants with HL, defined as having an older sibling with a clinical diagnosis of autism confirmed by the Social Communication Questionnaire^34^ and Autism Diagnostic Interview–Revised.^35^ Infants with LL were defined as having an older sibling with development within reference ranges or no siblings with autism at the time of enrollment. Participants were scanned at ages 6, 12, and 24 months, and the diagnostic outcome for autism was determined at age 24 months based on the Social Communication Questionnaire, Autism Diagnostic Interview–Revised, Autism Diagnostic Observation Schedule,^36^ and all available clinical information using the *Diagnostic and Statistical Manual of Mental Disorders* (Fourth Edition, Text Revision) checklist. Further information on clinical assessment and diagnosis has been published elsewhere.^37^

All participants included had successful brain scans at ages 12 and 24 months, acquired during natural sleep, and a diagnostic outcome at 24 months. In addition, approximately 80% of participants had a successful 6-month scan.

Full exclusion criteria for IBIS participants are described in detail elsewhere.^32^ Exclusionary factors included not having an older sibling, all types of obstructive hydrocephalus, seizure disorders at the time of enrollment (with the exception of febrile seizures), gestational diabetes, having diabetic siblings, cavernous hemangioma, pulmonary stenosis, and any other significant heart defects that required cardiological follow-up, thereby minimizing the potential confounding of vascular risk factors on PVS.

### MRI Acquisition

All brain scans were conducted during natural sleep on identical 3T Tim Trio scanners with a 12-channel head coil at 4 IBIS sites in the US.^32^ A 3-dimensional (3D) T1-weighted magnetization-prepared rapid acquisition gradient-echo scan (repetition time, 2400 ms; echo time, 3.16 ms; 160 sagittal slices; field of view [FOV], 256 mm; voxel size, 1 mm^3^) and 3D T2-weighted fast spin echo scan (repetition time, 3200 ms; echo time, 499 ms; 160 sagittal slices; FOV, 256 mm; voxel size, 1 mm^3^) were acquired in each participant. Preprocessing steps are described in eMethods in Supplement 1 and published elsewhere.^19^

### Identification of Enlarged PVS

The presence of enlarged PVS were visually identified during blinded radiological review by a board-certified neuroradiologist (R.C.M.) and confirmed through blind review by a second neuroradiologist (D.W.S). PVS were examined primarily using the T2-weighted scans, but the T1-weighted image was used in the rare instances when a T2-weighted scan was not acquired (23 scans [2.6%]). Each scan was rated as either having any visible enlarged PVS or no visible enlarged PVS (eMethods in Supplement 1).

### EA-CSF Volume Quantification

EA-CSF was segmented using an automated algorithm^19^ developed by our laboratory, which has been published previously^18,20^ (eMethods in Supplement 1). Unlike PVS enlargement, which could be completed by radiological inspection of only the T2-weighted scan, quantifying EA-CSF volume required both successful T1- and T2-weighted scans.

### Sleep Measure at School-Age Follow-Up

A subset of infants returned years later for a visit at school-age. At the school-age follow-up, children’s sleep characteristics were collected through the parent-reported Children’s Sleep Habits Questionnaire (CSHQ).^38^ The CSHQ delineates 8 sleep domains (eMethods in Supplement 1) that are then summed to generate an index of total sleep problems (range 33-99; with higher scores indicating greater sleep dysfunction). Based on previous studies that have observed an association between enlarged PVS and sleep disturbances in clinical^26,27^ and population-based samples,^28^ this sleep analysis focused on the night wakings subscale as an index of sleep disturbances. The CSHQ Night Wakings subscale is composed of 3 items that serve as an index for sleep disturbances in children (range, 3-9); this CSHQ subscale has been shown to be specifically correlated with sleep actigraphy.^39^ Additionally, the CSHQ total sleep problems score was used to measure overall sleep quality.

### Covariates

For multivariate models, covariates included age at scan, sex, and total cerebral volume (TCV). The groups were well-matched on age at each of the 6-, 12-, and 24-month time points, but precise age at scan was included to further control for any age variability. Sex was used as a covariate to test for effects of sex and to account for the greater proportion of males who developed autism. TCV was included as a covariate, as it has previously been shown to be associated with EA-CSF^18^ and to control for possible brain size differences between groups.^40,41^

### Statistical Analysis

Analyses were conducted using R software version 4.0.3 (R Project for Statistical Computing). All statistics are reported with a 2-sided *P* < .05 threshold. Data were analyzed from March 2021 through August 2022.

#### Primary Analysis

We used χ^2^ tests to test for group differences in the rates of PVS enlargement at ages 6, 12, and 24 months. Post hoc analyses for group differences were corrected for multiple comparisons (3 groups per time point) using false discovery rate (FDR) correction.^42^ To determine the extent to which the presence of enlarged PVS is associated with the likelihood that an infant with HL will receive an autism diagnosis, we examined odds ratios (ORs)^43^ in participants with HL who did vs did not receive an autism diagnosis. Following this primary analysis, the time point when enlarged PVS were most common across all groups was examined in follow-up analyses to evaluate the associations between enlarged PVS at this time point and other correlates of interest (ie, EA-CSF and sleep).

#### Secondary Analyses

We used a longitudinal mixed-effects model for repeated measures with unstructured covariance matrices was used to analyze the association between EA-CSF volume from ages 6 to 24 months and enlarged PVS. This statistical method is suitable for an unbalanced design and allows for missing values in a longitudinal study. Enlarged PVS were entered as an independent variable for all analyses. Sex and diagnostic group were added as covariates, and age at scan and TCV were added as time-dependent covariates. Effect of outliers was reduced through a winsorization technique^44^ (eMethods in Supplement 1). Furthermore, we explored whether the association of EA-CSF volume with enlarged PVS differed by groups by testing the interaction with diagnostic group.

Analysis of variance models were fit to test the association between enlarged PVS and school-age sleep problems, controlling for age at scan, sex, group, and TCV. Details on FDR correction can be found in the eMethods in Supplement 1. 

## Results

The total sample size was 311 participants (197 [63.3%] male) were included in analyses, with 3 outcome groups: 47 infants who were HL-positive (40 [85.1%] male), 180 infants who were HL-negative (102 [56.7%] male), and 84 infants who were LL-negative (55 [65.5%] male) at age 24 months. Age, sex, and number of scans per time point can be found in Table 1, while full demographics can be found in eTable 1 and eTable 2 in Supplement 1. In total, 870 scans were analyzed across the 3 time points, including 173 scans (19.9%) showing any visible enlarged PVS and 697 scans (80.1%) showing no visible enlarged PVS. EA-CSF volumes were available for 734 of 870 scans with PVS ratings (84.4%). CSHQ data at school-age were available for 109 of 311 infants with MRI data.

**Table 1.** Participant Characteristics by Diagnostic Outcome Group^a^

Characteristic	Mean (SD)			Test statistic	*P* value
	HL-positive	HL-negative	LL-negative		
**Age 6-24 mo visits (N = 311)**					
No.	47	180	84	NA	NA
Sex, No. (%)					
Male	40 (85.1)	102 (56.7)	55 (65.5)	14.56^b^	<.001
Female	7 (14.9)	78 (43.3)	29 (46.4		
Age, mo					
First MRI	6.5 (0.8)	6.6 (0.8)	6.7 (0.8)	0.50 (2,245)^c^	.61
Second MRI	12.7 (0.8)	12.6 (0.7)	12.6 (0.8)	0.49 (2,308)^c^	.61
Third MRI	24.7 (0.8)	24.7 (1.0)	24.7 (1.1)	0.06 (2,308)^c^	.95
**MRI scans (N = 870)**					
Total	129	495	246	NA	NA
6 mo (n = 248)	35	135	78	NA	NA
12 mo (n = 311)	47	180	84	NA	NA
24 mo (n = 311)	47	180	84	NA	NA
**School-age follow-up**					
No.	14	57	38	NA	NA
Sex, No. (%)					
Male	13 (92.9)	29 (50.9)	25 (65.8)	8.82^b^	.01
Female	1 (7.1)	28 (49.1)	13 (34.2)		
Age, mo	121.8 (13.8)	121.5 (15)	116.9 (13.1)	1.35 (2,106)^c^	.26
CSHQ Score					
Night wakings^d^	4.4 (1.7)	3.7 (1.1)	3.7 (1.4)	1.86 (2,106)	.16
Total sleep problems^e^	46.1 (10.2)	42.6 (7.5)	41.4 (7.6)	1.81 (2,106)^c^	.17

Abbreviations: CSHQ, Children’s Sleep Habits Questionnaire; HL-negative, higher likelihood of autism with no autism diagnosis; HL-positive, high likelihood of autism with an autism diagnosis; LL-negative, low likelihood of autism with no autism diagnosis; MRI, magnetic resonance imaging; NA, not applicable.

^a^ Data on maternal education, ethnicity, and household income can be found in eTable 1 and eTable 2 in Supplement 1.

^b^ Expressed as χ^2^_2_.

^c^ Expressed as F (*df*).

^d^ Range, 3 to 9; higher scores indicate more wakings.

^e^ Range, 33 to 99; higher scores indicate greater sleep dysfunction.

### Prevalence of Enlarged PVS Among Infants Who Develop Autism

**Figure 1.**
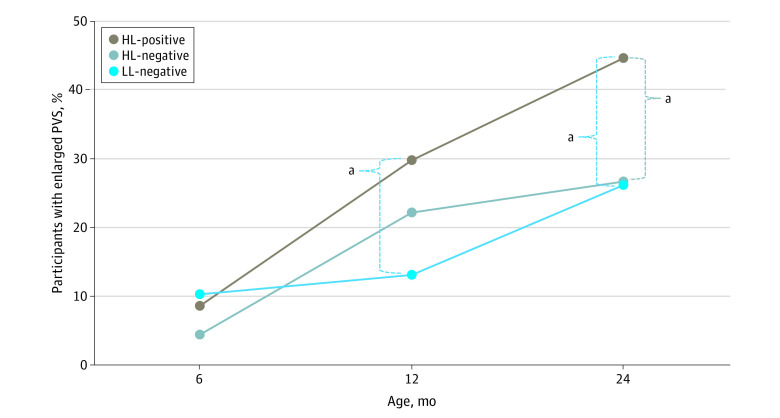
Presence of Enlarged Perivascular Spaces (PVS) at Ages 6 to 24 Months HL-negative indicates high likelihood of autism (ie, an older sibling with autism diagnosis) and no autism diagnosis at age 24 months; HL-positive, high likelihood of autism and autism diagnosis at age 24 months; and LL-negative, low likelihood of autism (ie, no older sibling with autism diagnosis) and no autism diagnosis at age 24 months. ^a^*P* < .05.

At age 6 months, enlarged PVS were observed in only 17 infants (6.9%), with no significant differences among groups (3 infants who were HL-positive [8.6%]; 6 infants who were HL-negative [4.4%]; 8 infants who were LL-negative [10.3%]; *P* = .25) (Table 2). At age 12 months, 14 infants who were HL-positive (29.8%) had enlarged PVS, compared with 11 infants were LL-negative (13.1%) (pairwise group comparison, *P* = .02) and 40 infants who were HL-negative (22.2%) (pairwise group comparison, *P* = .28). The overall effect of group was *P* = .06. At age 24 months, the overall group differences became more robust (overall effect of group, *P* = .04), with 21 infants who were HL-positive (44.7%) having enlarged PVS, which was greater than both the HL-negative group (48 infants [26.7%]) (*P* = .02) and the LL-negative group (22 infants [26.2%]) (*P* = .03) (Table 2). No significant differences in enlarged PVS were observed between the HL-negative and LL-negative groups at any time point (Table 2). Only post hoc pairwise comparisons at 24 months survived post hoc FDR correction at *q* = 0.05. Figure 1 presents the rates of enlarged PVS in each of the 3 groups over time.

**Table 2.** Presence of PVS Enlargement by Diagnostic Outcome Group

Time point, mo	Participants with enlarged PVS, No./total No. (%)			χ^2^_2_	*P* value	Full sample, No./total No. (%)
	HL-positive	HL-negative	LL-negative			
6	3/35 (8.6)	6/135 (4.4)	8/78 (10.3)	2.80	.25	17/248 (6.9)
12	14/47 (29.8)	40/180 (22.2)	11/84 (13.1)	5.53	.06	65/311 (20.9)
24	21/47 (44.7)	48/180 (26.7)	22/84 (26.2)	6.37	.04	91/311 (29.3)

Abbreviations: HL-negative, higher likelihood of autism with no autism diagnosis at 24 mo; HL-positive, high likelihood of autism with an autism diagnosis at 24 mo; LL-negative, low likelihood of autism with no autism diagnosis at 24 mo; PVS, perivascular space.

We conducted a parallel analysis to evaluate whether enlarged PVS was associated with increased likelihood of an autism diagnosis among infants with HL. The presence of enlarged PVS at 24 months was associated with a 2.22 times greater likelihood of autism diagnosis in children with HL (OR, 2.22; 95% CI, 1.44-4.31; *P* = .02). Of 69 infants with HL with enlarged PVS at 24 months, 21 (30.4%) received an autism diagnosis, compared with 26 of 132 of infants (19.7%) with HL without enlarged PVS.

### Association Between Enlarged PVS and EA-CSF Volume

**Figure 2.**
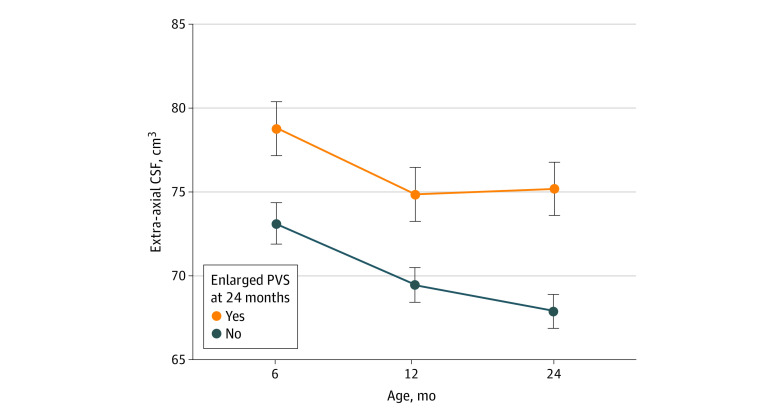
Association of Enlarged Perivascular Spaces (PVS) at 24 Months With Greater Extra-Axial Cerebrospinal Fluid (CSF) Volume at Ages 6 to 24 Months

Given that 24 months was the time point when enlarged PVS were most common in all groups, we explored whether infants who had enlarged PVS at 24 months had greater volumes of EA-CSF from ages 6 to 24 months. The full sample was split by either having or not having enlarged PVS at 24 months. The longitudinal mixed-effects model indicated that having enlarged PVS at 24 months of age was significantly associated with greater EA-CSF volumes from ages 6 to 24 months, even after controlling for diagnostic group, age, sex, and TCV (β = 4.64; 95% CI, 0.58-8.72; SE, 2.03; *F* = 9.79; *P* = .002, *d* = 0.27) (Figure 2). This association between greater EA-CSF volume from ages 6 to 24 months and enlarged PVS at 24 months was significant across all groups. In fact, there was no significant difference between either group with HL and the LL group (*F* = 0.26; *P* = .77), with the HL-positive group differing by 1.096 (SE, 2.98; *P* = .71; *d* = 0.04) and the HL-negative group differing by 1.325 (SE, 2.12; *P* = .53; *d* = 0.06). These small differences indicated that the association between EA-CSF volume and enlarged PVS was present across all diagnostic outcomes and was independent of family autism history. Sex was a significant covariate (β = 4.92; SE, 2.11; *F* = 11.95; *P* < .001; *d* = 0.28), with males generally having greater EA-CSF (*t* = 2.33; *P* = .02). TCV was not significantly associated with PVS (*β* = 0.00002; SE = 0.00001; *F* = 4.38; *P* = .06; *d* = 0.22). There was no significant association of age (β = 0.56; SE = 0.59; *F* = 2.50; *P* = .11; *d* = 0.11) and no significant PVS-by-age interaction (β = −0.09; SE = 0.10; *F* = 0.87; *P* = .35; *d* = 0.11), indicating that the increased EA-CSF volumes observed in infants with enlarged PVS was consistent across age.

### Associations With Later Sleep Problems

**Figure 3.**
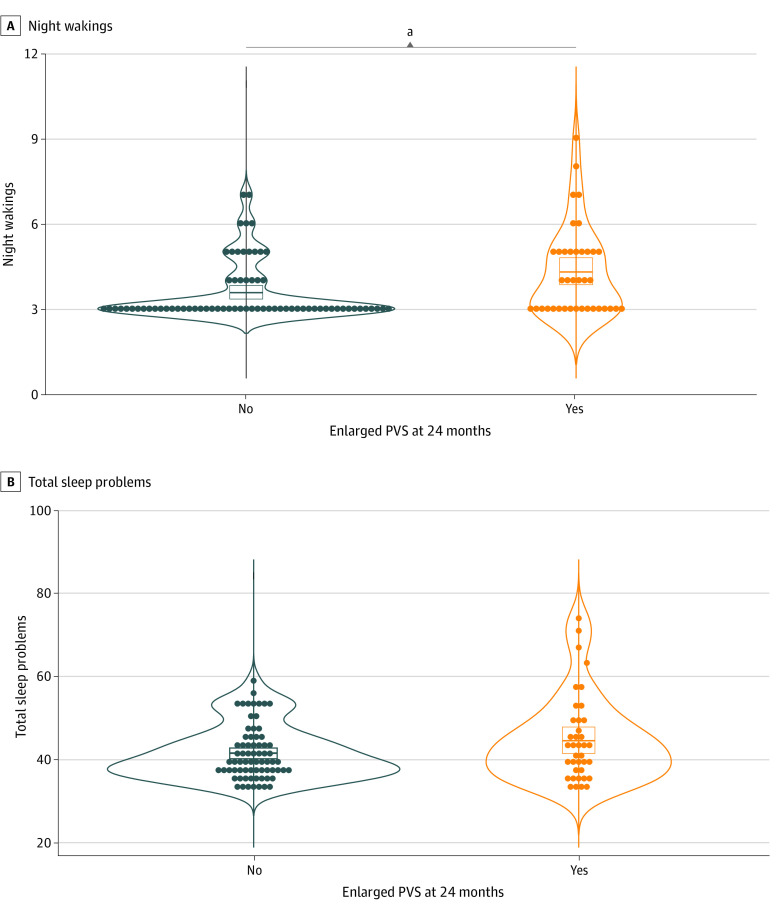
Association of Enlarged Perivascular Spaces (PVS) at Age 24 Months With Sleep Problems at School-Age Dots indicate individual data points; bar, mean; box, 95% CI. ^a^*P* < .01.

A subset of children returned approximately 8 years later for evaluation at school-age, thereby affording the opportunity to test whether early enlarged PVS were associated with long-term sleep problems. The subset characteristics and mean CSHQ scores are reported in Table 1. We found that infants with enlarged PVS at age 24 months, compared with those without enlarged PVS, had higher rates of night wakings at school-age (mean [SE] subscale score, 4.29 [0.24] vs 3.56 [0.12]; *F* = 7.76; η^2^ = 0.08; 95% CI, 0.22-1.24; *P* = .006, FDR-corrected *P* = .01) (Figure 3A). There were no significant associations with age at scan (*F* = 0.69; η^2^ = 0.007; *P* = .40), sex (*F* = 1.07; η^2^ = 0.01; *P* = .30), diagnostic group (*F* = 2.12; η^2^ = 0.04; *P* = .13), and TCV at age 24 months (*F* = 1.35; η^2^ = 0.01; *P* = .25). Since neither diagnostic group nor the PVS × diagnostic group interaction (*F* = 0.33; η^2^ = 0.007; *P* = .72) were significant in the model, these results suggest that having enlarged PVS at age 24 months was associated with more frequent night disturbances regardless of familial likelihood or diagnosis of autism. These results remained relatively unchanged when examined as a categorical trend analysis (eAppendix in Supplement 1), as well as when EA-CSF was entered as a covariate (*F* = 5.30; η^2^ = 0.059; *P* = .02), indicating that enlarged PVS might have a unique association with sleep disturbances. For the sake of completeness, we evaluated the association with the more general index of total sleep problems and found that children with enlarged PVS at age 24 months, compared with those without enlarge PVS, did not have statistically significantly increased scores on the total index of overall sleep problems after controlling for age, sex, group, and TCV (mean [SE] total score, 44.58 [1.53] vs 41.59 [0.78]; *F* = 3.01, η^2^ = 0.031; 95% CI, −0.37 to 6.07; *P* = .09) (Figure 3B).

## Discussion

### Rate of Enlarged PVS in Autism

To our knowledge, this cohort study was the first to examine PVS during infancy in association with autism. Infants who were HL-positive had significantly higher rates of enlarged PVS than infants not diagnosed with autism, both with and without a family history of autism. Group differences in enlarged PVS became pronounced by age 24 months, with nearly half of infants diagnosed with autism having enlarged PVS. At age 24 months, enlarged PVS were found in 45% of participants who were HL-positive, compared with only 26% of infants who were HL-negative and infants who were LL-negative. The rate of enlarged PVS in individuals aged 24 months with autism was nearly identical to the previously reported 44% rate of enlarged PVS in a smaller sample of children with autism aged 7 to 18 years.^16^ Additionally, the presence of enlarged PVS at 24 months was associated with a 2.2-fold greater likelihood of autism diagnosis among children with HL. Our findings suggest that enlarged PVS at age 24 months may be a relatively common neurological feature of autism, occurring earlier than previously recognized, and detectable by MRI at an age that precedes the typical age of autism diagnosis.^45^

### PVS, EA-CSF, and the Glymphatic System

The brainwide network of perivascular spaces in the glymphatic system^1^ plays a crucial role in the clearance of metabolic waste and fluid from the brain: EA-CSF flows into the PVS in the brain parenchyma where a rapid exchange of CSF and interstitial fluid occurs.^8,46^ Through effective circulation and absorption, EA-CSF volume decreases over the first 2 years of life.^18,19,47^ If there is a deficit in absorption, it is hypothesized that the accumulation of EA-CSF, which was previously been observed in autism,^18,19,20^ might contribute to a later dilation of PVS as cranial sutures close and thus force the CSF into the parenchyma. Our findings supported this hypothesis: increased EA-CSF volume from ages 6 to 24 months was associated with enlarged PVS at age 24 months across all infants, regardless of likelihood or diagnosis of autism. This finding suggests that the association between PVS and EA-CSF volume could be a physiological process of the developing glymphatic system in the first 2 years of life.

### Glymphatic System and Sleep

Recent discoveries have indicated that sleep and CSF circulation within the glymphatic system are linked. Nedergaard and colleagues^29^ were the first to show that uptake of CSF into PVS is increased during sleep in rodents, and there have been a few studies showing a similar pattern in humans.^48,49^ Additionally, there has been increasing evidence indicating that PVS dilation in adults is linked with disrupted sleep.^26,27,28^ It is believed that disrupted sleep reduces PVS flushing, resulting in impaired clearance of metabolic byproducts from the brain. In this study, we examined PVS in association with sleep behaviors in school-aged children and found that enlarged PVS at age 24 months were associated with more frequent night wakings at school age. Furthermore, enlarged PVS had a unique association with night wakings, which remained significant even when controlling for different levels of EA-CSF volume. These findings provide initial evidence that early glymphatic system markers in infancy could precede later sleep problems at school age.

### Strengths

To our knowledge, this is the largest sample of infants who were LL-negative to be examined for PVS anomalies and the first study done in infants with HL. While there is converging evidence from rodent^1^ and human adult studies^50,51^ to suggest that CSF between the subarachnoid space (ie, EA-CSF) and PVS are anatomically and physiologically linked, these studies used invasive fluorescent tracer injections that are not suitable for research studies in children. Our study involved 2 noninvasive measures of glymphatic function during infancy by using structural MRI collected during natural sleep. This study design allowed us to characterize the longitudinal, dynamic association between glymphatic components during the sensitive developmental period of human infancy. Furthermore, given that this study cohort is one of longest running longitudinal studies to examine brain development in autism,^19,32,33,40,52,53,54,55,56^ we had an opportunity to examine whether enlarged PVS during infancy were related to sleep outcomes 8 years later.

### Future Directions

Our study relied on qualitative PVS characterization by expert neuroradiological review because this is the standard in the field of pediatric neuroimaging and because quantitative PVS methods have not been validated in infants, given low white and gray matter contrast in the first year of life. In the future, automated quantification of PVS volume across different regions of the brain^57,58^ would allow for the examination of PVS on a continuum and provide further important insights on the morphology, location, and change over time in PVS during infancy. The addition of a dynamic, physiological measure of CSF flow, such as phase-contrast MRI^59^ or newer state-of-the-art flow sequences,^60,61,62^ would allow us to examine outcomes associated with CSF circulation during typical and aberrant brain development. Additions of direct sleep monitoring, through electroencephalograms and polysomnography, could offer insights into sleep regulation mechanisms underlying night wakings.^63^ Future studies should also examine disorder-specificity to see whether other developmental disorders, such as Down syndrome or fragile X syndrome, also have increased rates of PVS enlargement outside reference ranges. Lastly, future studies will be needed to determine whether enlarged PVS is developmentally transient or persistent into adulthood. This will be key in elucidating whether early life glymphatic dysfunction is linked to early neurodegeneration, which occurs at higher rates in autism and other neurodevelopmental disorders.^64,65^

### Limitations

This study has some limitations. While our sample was larger than what exists in the literature, we may have been underpowered to directly compare whether the groups differed in the association between enlarged PVS and later sleep, given that we only had school-age sleep data for 14 participants in the HL-positive group. Instead, we controlled for group in the model. Additionally, sleep data were limited to the parent-reported CSHQ at 1 time point (school-age), whereas more objective assessment of night wakings could be gained by sleep actigraphy or sleep sensor mats. At school-age, parents may not be aware every time their child awakens during the night unless the child wakes them up. Therefore, the night wakings scale could represent a conservative estimate and provide room for type II errors but fewer type I errors. The temporal order between glymphatic features (enlarged PVS and EA-CSF) and sleep problems remains unclear, since sleep data were not collected longitudinally. Our group previously reported that in children with autism ages 2 to 4 years, elevated EA-CSF volumes were associated with increased rates of concurrent sleep problems,^20^ but additional studies are needed to examine sleep behaviors in infancy to determine whether sleep problems precede or follow glymphatic function outside reference ranges.

## Conclusions

This cohort study found that infants who were HL-positive had significantly increased rates of enlarged PVS by age 24 months compared with infants who were HL-negative and LL-negative. Results suggest that enlarged PVS could be a risk factor associated with developing autism, but more studies are needed to determine whether it could be a stratifying marker to differentiate infants with HL who will or will not develop autism themselves. Elevated EA-CSF volumes were observed from age 6 to 24 months in participants who had enlarged PVS by age 24 months, supporting the hypothesis that an accumulation of EA-CSF could be linked with PVS dilation and might provide crucial insight into an underlying mechanism in glymphatic function during infancy. In a subset of participants with school-age sleep data, enlarged PVS at 24 months was associated with higher frequency of night wakings at school-age. Taken together, these results support that enlarged PVS during infancy could be indicative of developmental delays or disorders and have implications for later sleep problems.

Studies of glymphatic function to date have largely focused on aging and neurodegeneration, and this study offers the first examination of the development and function of the glymphatic system in infancy using noninvasive imaging. Beyond neurodegenerative disorders, examining glymphatic function in neurodevelopmental disorders could have a significant impact on early diagnosis and clinical outcomes for children with autism.

## Notes

Supplement 1. **eMethods.** Supplementary Methods **eAppendix.** PVS and Sleep: Categorical Trend Analysis **eTable 1.** Participant Demographics by Diagnostic Outcome Group, 6-24 Months **eTable 2.** Participant Demographics by Diagnostic Outcome Group, School-Age Follow-Up

Supplement 2.
Members of the Infant Brain Imaging Study (IBIS) Network

Supplement 3.
Data Sharing Statement
