# Supplementary material for: Enlarged Perivascular Spaces in Infancy and Autism Diagnosis, Cerebrospinal Fluid Volume, and Later Sleep Problems
Source: JAMA Netw Open. 2023 Dec 19;6(12):e2348341. doi: 10.1001/jamanetworkopen.2023.48341 (PMC10731509; doi:10.1001/jamanetworkopen.2023.48341)
Supplement: Supplement 1. — eMethods. Supplementary Methods eAppendix. PVS and Sleep: Categorical Trend Analysis eTable 1. Participant Demographics by Diagnostic Outcome Group, 6-24 Months eTable 2. Participant Demographics by Diagnostic Outcome Group, School-Age Follow-Up [file jamanetwopen-e2348341-s001.pdf]

Supplemental Online Content

Garic D, McKinstry RC, Rutsohn J, et al; for Infant Brain Imaging Study (IBIS) Network. Enlarged perivascular spaces in infancy and autism diagnosis, cerebrospinal fluid volume, and later sleep problems. *JAMA Netw Open*. 2023;6(12):e2348341. doi:10.1001/jamanetworkopen.2023.48341

eMethods. Supplementary Methods
eAppendix. PVS and Sleep: Categorical Trend Analysis
eTable 1. Participant Demographics by Diagnostic Outcome Group, 6-24 Months
eTable 2. Participant Demographics by Diagnostic Outcome Group, School-Age Follow-Up
eReferences.

This supplemental material has been provided by the authors to give readers additional information about their work.

© 2023 Garic D et al. *JAMA Network Open.*

eMethods. Supplementary Methods

MRI Preprocessing

As described in Shen et al.[1], all images were preprocessed with distortion correction, mutual registration, transformation to stereotactic space, tissue segmentation (white, gray, CSF), and corrected for geometric distortions and intensity non-uniformity. Specifically, mutual information registration[2] was used to perform linear, rigid registration of the T2-weighted images to corresponding T1-weighted images. Next, both the T1- and T2-weighted images were transformed to stereotactic space on the T1 registration. Skull extraction was completed using FSL's Brain Extraction Tool[3], with a "majority voting approach" between the T1 and T2 atlas masks and T1 and T2 images jointly. All scans were blindly reviewed to ensure only skull was removed and the entire intracranial and subarachnoid spaces were left intact. All corrected and skull-stripped T1 and T2 images were used as input for the AutoSeg pipeline[4] to obtain white matter, gray matter and CSF tissue segmentation. Lastly, two study-specific atlas templates were created to maximize age-specific tissue segmentation. Atlases were derived from 104 training images via joint deformable registration[5] and then applied to 6 month and 12-24 month data sets.

Identification of Enlarged Perivascular Spaces (ePVS)

The radiologists first determined whether there was visible ePVS (N=173 out of 870, 19.89%) or not (N=697 of 870 scans, 80.11%). If there were visible ePVS, then a severity rating was applied on a 1-4 scale: 1 = minimal enlargement (i.e., defined as fewer than five small, linear T2 hyperintensities or one or two larger foci); 2 = mild enlargement (i.e., defined as 5-10 small, linear or 3-5 larger, rounded hyperintensities); 3 = moderate enlargement (i.e., defined as greater than 10 linear or 5–10 larger, rounded hyperintensities); 4 = marked enlargement (which involved pathological number, size, and location of the hyperintensities). To remain consistency for the entire dataset, the first neuroradiologist reviewed all 870 MRI scans for ePVS; the second neuroradiologist confirmed these for ePVS.

Of the scans that had visible ePVS comprising scores 1-4 (173 of 870 scans), nearly all of these (97.7%) had scores of 1 or 2, and the remaining 2.3% had scores of 3 or 4. Given the lack of range in severity scores, the severity scores were not used in the analysis. Therefore, we collapsed scores 1-4, and PVS enlargement was analyzed as a binary classification: no visible PVS enlargement (score of 0); or PVS enlargement (comprising scores of 1-4). Binary classification for qualitatively-rated PVS is a common practice in the field[6-10].

Extra-Axial Cerebrospinal Fluid (EA-CSF) Volume Quantification

EA-CSF was segmented using an automated algorithm[1] based on our previous manual segmentation procedure[11,12]. A mask created from the study atlas templates was used to remove the ventricles and cisterns to isolate CSF in the subarachnoid space. To remain consistent with the manual segmentation previously used, we defined a ventral boundary at the horizontal plane of the anterior-posterior commissure, thereby making the dorsal subarachnoid space the region of interest. All EA-CSF segmentations were visually inspected by a blinded experimenter, and a subset (n = 52) were compared with both manual and automated segmentation to ensure consistency (ICC = .80).

Sleep Measure at School-age Follow-Up

At the school-age follow-up, children's sleep characteristics were collected through the parent-reported Children's Sleep Habits Questionnaire (CSHQ)[13]. The measure delineates eight sleep domains (bedtime resistance, sleep onset delay, sleep duration, sleep anxiety, night wakings, parasomnias, sleep-disordered breathing, daytime sleepiness) and after removing duplicate questions, 33 items were used to calculate an index of total sleep problems. Higher scores indicate greater sleep dysfunction. Owens et al. have demonstrated that the CSHQ has acceptable internal consistency in community and clinical samples (α =.68 and .78, respectively), test-retest reliability (r =.62 and .79) and was able to differentiate between children with and without sleep disorders with sensitivity of .80 and specificity of .72. While originally designed to assess sleep problems in

© 2023 Garic D et al. *JAMA Network Open.*

typically developing children, the CSHQ is one of the most widely used sleep questionnaires for samples of children with autism and has been consistent with actigraphy and polysomnography results[14-16]. Our primary sleep measure of interest was night wakings (three items: frequency of the child waking up once a night, multiple times a night, and moving to another person's bed at night) for two reasons: to our particular interest in the relationship between CSF dynamics and components of sleep disturbance, and because this sub-scale has been shown to be the only sub-scale that is significantly correlated to actigraphy results[17]. For the purposes of this study, we included all CSHQ assessments that were completed in full at the time of the analysis, which resulted in a sample size of 109 children (109 / 311 = 35%) (Table 1).

Correction for Multiple Comparisons

For the primary analysis examining group differences in enlarged PVS from 6-24 months of age, post-hoc analyses for group differences were corrected for multiple comparisons (3 groups per time point) using False Discovery Rate (FDR) correction[18]. Only post-hoc pairwise comparisons at 24 months survived post-hoc FDR correction at q=.05.

Correction for multiple comparisons were not deemed necessary for the relationship between PVS and EA-CSF. This analysis was examined in one longitudinal mixed effects model, and therefore there were no multiple comparisons.

FDR correction for two comparisons (CSHQ Night Wakings scale and CSHQ Total Score) was conducted for the associations between infant PVS and school-age sleep problems analyses. Only Night Wakings was significant (F=7.76, η^2=0.08, 95% CI=0.22-1.24, p=0.006, FDR-corrected p=0.01).

Outlier Correction

Outliers in EA-CSF volume could potentially be caused by either individual differences or segmentation overestimation, therefore values were clipped using a conservative 95% Winsorization procedure[19] to reduce the effect of extreme values. Winsorization was conducted in R using DescTools (v. 0.99.43, https://cran.r-project.org/package=DescTools). This procedure identified 12 EA-CSF volume outliers (out of 216, 5.5%) at 6 months of age (2 HL-positive, 6 HL-negative, 4 LL-negative), 14 outliers (out of 261, 5.4%) at 12 months of age (3 HL-positive, 4 HL-negative, 7 LL-negative), and 14 outliers (out of 257, 5.5%) at 24 months of age (2 HL-positive, 7 HL-negative, 5 LL-negative). *Abbreviations:* HL-positive: infants at high familial likelihood to develop autism who went on to develop autism themselves; HL-negative: high familial likelihood who did not develop autism; LL-negative: low familial likelihood and did not develop autism.

Resulting Sample

The resulting sample had a wide range of family income, which was coded on an 8-point ordinal scale (1 = less than $25,000, 8 = over $200,000, M = 4.72, SD = 1.77), and mothers were primarily college educated (coded on a 6-point ordinal scale with 1 = some high school, 6 = graduate degree, M = 4.26, SD = 1.30). Participants were primarily White (85.67%) and non-Hispanic (94.12%). The full demographic breakdown of the full Infant Brain Imaging Study (IBIS) sample is described in previous studies[20]. We did not find significant group differences on family income (p = .834), race (p = .413), ethnicity (p = .369), or on age of scan acquisition at any time point (p = .61-.95). Significant group differences were observed in maternal education (F = 4.91, p = .008), with the LL-negative sample indicating higher maternal education than the other two groups. Maternal education did not play a significant role (p = 0.88) in the PVS and EA-CSF models and the results remained unchanged when added.

Demographics in the school-age follow-up sample stayed relatively consistent to the full sample. No significant group differences were observed in maternal education (p = 0.101), race (p = .579), ethnicity (p = .757), or family income (p = .285).

© 2023 Garic D et al. *JAMA Network Open.*

eAppendix. PVS and Sleep: Categorical Trend Analysis
Since the CSHQ scores for Night Wakings were on a 3–9-point scale, we also ran the model with categorical trend analysis. The PVS-sleep results remained largely consistent, with a strong association between 24-month PVS and CSHQ night wakings (z = -2.8242, p = .0047). This was not run with total score (range 33-99) due some cells having <5 given the asymptotic assumptions of our sample.

© 2023 Garic D et al. *JAMA Network Open.*

eTable 1: Participant Demographics by Diagnostic Outcome Group; 6-24 Months

	Lower Likelihood Negative	Higher Likelihood Negative	Higher Likelihood Positive	Test Statistic[a]
n at 6-24 Month Visits	84	180	47	
**Maternal Education; M (SD)**	4.65 (1.26)	4.12 (1.28)	4.11 (1.33)	$F_{2,291}$ **= 4.91, *p* = .008**
1) Some high school	*1*	*2*	*0*	
2) High school graduate	*1*	*12*	*4*	
3) Some college	*11*	*40*	*12*	
4) College graduate	*32*	*70*	*15*	
5) Some grad school	*1*	*4*	*1*	
6) Graduate degree	*33*	*43*	*12*	
NA) Not answered	*5*	*9*	*3*	
**Race**				$F_{2,304}$ **= 0.89, *p* = .413**
Asian	*1*	*3*	*0*	
Black/African American	*4*	*3*	*0*	
White	*68*	*154*	*41*	
More than one race	*8*	*19*	*6*	
Not answered	*3*	*1*	*0*	
**Ethnicity**				χ^2_2 **= 1.99, *p* = .369**
Hispanic	*2*	*14*	*2*	
Non-Hispanic	*79*	*165*	*44*	
Not answered	*3*	*1*	*1*	
**Household Income:**	4.78 (1.61)	4.75 (1.87)	4.58 (1.71)	$F_{2,277}$ **= 0.18, *p* = .834**
1) Less than 25k	*3*	*8*	*1*	
2) 25k - 35k	*3*	*14*	*6*	
3) 35k - 50k	*9*	*18*	*3*	
4) 50k – 75k	*18*	*32*	*10*	
5) 75k – 100k	*14*	*30*	*10*	
6) 100k – 150k	*21*	*31*	*8*	
7) 150k – 200k	*5*	*14*	*3*	
8) Over 200k	*3*	*14*	*2*	
NA) Not answered	*8*	*19*	*4*	

Data represents mean and standard deviation in parentheses.
F, female; M, male; MRI, magnetic resonance imaging.
[a] Test statistic, degrees of freedom, and *p* value of chi-square for sex and omnibus analysis of variance for age.

© 2023 Garic D et al. *JAMA Network Open.*

eTable 2: Participant Demographics by Diagnostic Outcome Group; School-Age Follow-Up

	Lower Likelihood Negative	Higher Likelihood Negative	Higher Likelihood Positive	Test Statistic[a]
n at School-Age Follow-Up	38	57	14	
**Maternal Education; M (SD)**	4.76 (1.20)	4.18 (1.32)	4.29 (1.49)	**$F_{2,105}$ = 2.34, *p* = .101**
1) Some high school	*0*	*1*	*0*	
2) High school graduate	*1*	*3*	*2*	
3) Some college	*3*	*13*	*2*	
4) College graduate	*17*	*23*	*5*	
5) Some grad school	*0*	*0*	*0*	
6) Graduate degree	*17*	*16*	*5*	
NA) Not answered	*0*	*1*	*0*	
**Race**				**$F_{2,105}$ = 0.55, *p* = .579**
Asian	*1*	*1*	*0*	
Black/African American	*1*	*0*	*0*	
White	*34*	*50*	*13*	
More than one race	*2*	*5*	*1*	
Not answered	*0*	*1*	*0*	
**Ethnicity**				**χ^2_2 = 0.56, *p* = .757**
Hispanic	*1*	*3*	*0*	
Non-Hispanic	*37*	*53*	*13*	
Not answered	*0*	*1*	*1*	
**Household Income:**	4.81 (1.69)	4.78 (1.7)	4 (1.47)	**$F_{2,100}$ = 1.27, *p* = .285**
1) Less than 25k	*2*	*1*	*0*	
2) 25k - 35k	*1*	*4*	*3*	
3) 35k - 50k	*3*	*8*	*1*	
4) 50k – 75k	*10*	*11*	*4*	
5) 75k – 100k	*7*	*11*	*4*	
6) 100k – 150k	*8*	*10*	*0*	
7) 150k – 200k	*3*	*6*	*1*	
8) Over 200k	*2*	*3*	*0*	
NA) Not answered	*2*	*3*	1	

Data represents mean and standard deviation in parentheses.
F, female; M, male; MRI, magnetic resonance imaging.
[a] Test statistic, degrees of freedom, and *p* value of chi-square for sex and omnibus analysis of variance for age.

© 2023 Garic D et al. *JAMA Network Open.*

eReferences.

1. Shen MD, Kim SH, McKinstry RC, et al. Increased Extra-axial Cerebrospinal Fluid in High-Risk Infants Who Later Develop Autism. *Biol Psychiatry.* 2017;82(3):186-193.
2. Collins DL, Neelin P, Peters TM, Evans AC. Automatic 3D intersubject registration of MR volumetric data in standardized Talairach space. *J Comput Assist Tomogr.* 1994;18(2):192-205.
3. Smith SM. Fast robust automated brain extraction. *Hum Brain Mapp.* 2002;17(3):143-155.
4. Gouttard S, Styner M, Joshi S, Smith R, Cody Hazlett H, Gerig G. *Subcortical structure segmentation using probabilistic atlas priors.* Vol 6512: SPIE; 2007.
5. Coupe P, Manjon JV, Fonov V, Pruessner J, Robles M, Collins DL. Patch-based segmentation using expert priors: application to hippocampus and ventricle segmentation. *Neuroimage.* 2011;54(2):940-954.
6. Jeong SH, Cha J, Park M, et al. Association of Enlarged Perivascular Spaces With Amyloid Burden and Cognitive Decline in Alzheimer Disease Continuum. *Neurology.* 2022.
7. Zeegers M, Van Der Grond J, Durston S, et al. Radiological findings in autistic and developmentally delayed children. *Brain Dev.* 2006;28(8):495-499.
8. Rollins NK, Deline C, Morriss MC. Prevalence and clinical significance of dilated Virchow-Robin spaces in childhood. *Radiology.* 1993;189(1):53-57.
9. Song TJ, Park JH, Choi KH, et al. Moderate-to-severe obstructive sleep apnea is associated with cerebral small vessel disease. *Sleep Med.* 2017;30:36-42.
10. Del Brutto OH, Mera RM, Del Brutto VJ, Castillo PR. Enlarged basal ganglia perivascular spaces and sleep parameters. A population-based study. *Clin Neurol Neurosurg.* 2019;182:53-57.
11. Shen MD, Nordahl CW, Young GS, et al. Early brain enlargement and elevated extra-axial fluid in infants who develop autism spectrum disorder. *Brain.* 2013;136(Pt 9):2825-2835.
12. Shen MD, Nordahl CW, Li DD, et al. Extra-axial cerebrospinal fluid in high-risk and normal-risk children with autism aged 2-4 years: a case-control study. *Lancet Psychiatry.* 2018;5(11):895-904.
13. Owens JA, Spirito A, McGuinn M. The Children's Sleep Habits Questionnaire (CSHQ): psychometric properties of a survey instrument for school-aged children. *Sleep.* 2000;23(8):1043-1051.
14. Souders MC, Mason TB, Valladares O, et al. Sleep behaviors and sleep quality in children with autism spectrum disorders. *Sleep.* 2009;32(12):1566-1578.
15. Malow BA, Marzec ML, McGrew SG, Wang L, Henderson LM, Stone WL. Characterizing sleep in children with autism spectrum disorders: a multidimensional approach. *Sleep.* 2006;29(12):1563-1571.
16. Reed HE, McGrew SG, Artibee K, et al. Parent-based sleep education workshops in autism. *J Child Neurol.* 2009;24(8):936-945.
17. Markovich AN, Gendron MA, Corkum PV. Validating the Children's Sleep Habits Questionnaire Against Polysomnography and Actigraphy in School-Aged Children. *Front Psychiatry.* 2014;5:188.
18. Benjamini Y, Hochberg Y. Controlling the False Discovery Rate: A Practical and Powerful Approach to Multiple Testing. *Journal of the Royal Statistical Society Series B (Methodological).* 1995;57(1):289-300.
19. Wilcox RR, Keselman HJ. Modern robust data analysis methods: measures of central tendency. *Psychol Methods.* 2003;8(3):254-274.
20. Hazlett HC, Gu H, Munsell BC, et al. Early brain development in infants at high risk for autism spectrum disorder. *Nature.* 2017;542(7641):348-351.

© 2023 Garic D et al. *JAMA Network Open.*
