# Supplementary material for: Enlarged Perivascular Spaces in Infancy and Autism Diagnosis, Cerebrospinal Fluid Volume, and Later Sleep Problems
Source: JAMA Netw Open. 2023 Dec 19;6(12):e2348341. doi: 10.1001/jamanetworkopen.2023.48341 (PMC10731509; doi:10.1001/jamanetworkopen.2023.48341)
Supplement: Supplement 2. — Members of the Infant Brain Imaging Study (IBIS) Network [file jamanetwopen-e2348341-s002.pdf]

Supplemental Online Content: Nonauthor Collaborators
*First name, last name, and suffix (if applicable) are required and will appear in PubMed.

*Group Name(s): Infant Brain Imaging Study (IBIS) Network							
***First Name and Middle Initial(s)**	***Last Name**	***Suffix (eg, Jr, III)**	Academic Degrees	Institution	Location (city, state/province, country)	Role or Contribution, eg, chair, principal investigator	Group (if more than 1 Group listed in the byline) and/or Subgroup (eg, Steering Committee)
Lonnie	Zwaigenbaum		MD	University of Alberta	Edmonton, Alberta, Canada		
Alan C.	Evans		PhD	Montreal Neurological Institute	Montreal, Quebec, Canada		
John R.	Pruett	Jr.	MD	Washington University School of Medicine in St. Louis	St. Louis, MO, USA		
Guido	Gerig		PhD	New York University	New York, NY, USA		
Meghan R.	Swanson		PhD	University of Texas at Dallas	Richardson, TX, USA		
Jed T.	Elison		PhD	University of Minnesota	Minneapolis, MN, USA		
Dennis	Shaw		MD	University of Washington	Seattle, WA, USA		
D. Louis	Collins		PhD	Montreal Neurological Institute	Montreal, Quebec, Canada		
Jessica B.	Girault		PhD	University of North Carolina at Chapel Hill	Chapel Hill, NC, USA		
J. Chad	Chappell		MA	University of North Carolina at Chapel Hill	Chapel Hill, NC, USA		
Kinh	Truong		PhD	University of North Carolina at Chapel Hill	Chapel Hill, NC, USA		
Santiago	Torres		PhD	Montreal Neurological Institute	Montreal, Quebec, Canada		
