# Supplementary material for: Enlarged Perivascular Spaces in Infancy and Autism Diagnosis, Cerebrospinal Fluid Volume, and Later Sleep Problems
Source: JAMA Netw Open. 2023 Dec 19;6(12):e2348341. doi: 10.1001/jamanetworkopen.2023.48341 (PMC10731509; doi:10.1001/jamanetworkopen.2023.48341)
Supplement: Supplement 3. — Data Sharing Statement [file jamanetwopen-e2348341-s003.pdf]

Data Sharing Statement

Garic. Enlarged Perivascular Spaces in Infancy and Autism Diagnosis, Cerebrospinal Fluid Volume, and Later Sleep Problems. *JAMA Netw Open*. Published December 19, 2023. doi:10.1001/jamanetworkopen.2023.48341

Data
Data available: Yes
Data types: Deidentified participant data
How to access data: Data will be available upon reasonable request to the corresponding author (dea_garic@med.unc.edu)
When available: With publication

Supporting Documents
Document types: None

Additional Information
Who can access the data: Researchers whose proposed use of the data has been approved
Types of analyses: For a specified purpose
Mechanisms of data availability: After approval of a proposal
